# Supplementary material for: Enabling and constraining successful reablement: Individual and neighbourhood factors
Source: PLoS One. 2020 Sep 4;15(9):e0237432. doi: 10.1371/journal.pone.0237432 (PMC7473582; doi:10.1371/journal.pone.0237432)
Supplement: S1 Table — (PDF) [file pone.0237432.s001.pdf]

Reablement Service Measurement Tool (SMT)						
**Domain**	**Score**	**4**	**3**	**2**	**1**	**0**
**Mobility**	Indoors	Independent without aid	Independent with aid	Physical minimum assistance	Frequent falls/ max assistance	Unable
	Outdoors	Independent without aid	Independent with aid	Minimal physical assistance	Maximum physical assistance	Unable
	Steps/stairs	Independent	Some difficulty slow but safe	Very slow great physical exertion	At risk	Unable
	Transport	Able to access all forms of transport independently	Able to access all forms of transport with min assistance	Able to access all forms of transport with max assistance	Able to access one form of transport with assistance	Unable
**Transfers**	Bed	Independent	Slow but safe	Very slow with great physical exertion	Physical assistance required	Unable
	Chair	Independent	Slow but safe	Very slow with great physical exertion	Physical assistance required	Unable
	Toilet/commode	Independent	Slow but safe	Very slow with great physical exertion	Physical assistance required	Unable
	Bath or shower	Independent	Slow but safe	Very slow with great physical exertion	Physical assistance required	Unable
	Dressing	Independent	Prompts required	Prompts and physical assistance required	Full physical assistance required	Unable
	Undressing	Independent	Prompts required	Prompts and physical assistance required	Full physical assistance required	Unable

**Personal Care Skills**	Washing	Independent	Prompts required	Prompts and physical assistance required	Full physical assistance required	Unable
	Bathing or showering	Independent	Prompts required	Prompts and physical assistance required	Full physical assistance required	Unable
	Eating	Independent	Some difficulty	Great difficulty	Assistance required	Unable
	Drinking	Independent	Some difficulty	Great difficulty	Assistance required	Unable
	Toilet hygiene	Independent	Some difficulty	Great difficulty	Physical assistance required	Unable
**Home Skills**	Meal preparation/ cooking	Independent	Able with prompts	Able with prompts and min assistance	Requires physical assistance	Unable
	Washing up/clearing away	Independent	Able with prompts	Able with prompts and min assistance	Requires physical assistance	Unable
	Shopping	Independent	Able with prompts	Able with prompts and min assistance	Requires physical assistance	Unable
	Hoovering	Independent	Able with prompts	Able with prompts and min assistance	Requires physical assistance	Unable
	Bathroom cleaning	Independent	Able with prompts	Able with prompts and min assistance	Requires physical assistance	Unable
	Change bed linen	Independent	Able with prompts	Able with prompts and min assistance	Requires physical assistance	Unable
	Laundry	Independent	Able with prompts	Able with prompts and min assistance	Requires physical assistance	Unable

	Ironing	Independent	Able with prompts	Able with prompts and min assistance	Requires physical assistance	Unable / unsafe
**Sensory**	Speech	No speech problems	Slight difficulty	Significant difficulty	Uses non-verbal communicati on	Unable
	Sight (with glasses)	Satisfactory	Slight difficulty	Significant difficulty	Registered Partially sighted	Registered Blind
	Hearing (with aid)	Satisfactory	Slight impairment	Registered deaf with speech	Registered deaf without speech with sign	Registered deaf without speech without sign
	Sensation fingers/feet	No impairment of sensation	Slight impairment of sensation	Significant impairment of sensation	Significant impairment of sensation with risk	Neglect to affected area
**Health & Understan ding**	Can manage own medication	Independent	Manage with verbal prompts	Manage with directed prompts	Requires physical assistance	Unable
	Home environment control	Independent	Manage with verbal prompts	Manage with directed pro mpts	Requires physical assistance	Unable
	Confusion	No confusion	Mild confusion not at risk	Moderate confusion risks managed	At risk	Unsuitable for reablement
	Memory	No difficulties	Slight difficulty	Occasional prompts required	Frequent prompt and repetition	Extreme memory loss
	Attention/ concentratio n	Maintains attention/ concentratio n	Able to maintain attention on one task	Requires prompts to maintain attention to task	Requires prompts and assistance	Unable
	Motivation	Fully motivated	Requires encouragem ent	Requires directed verbal prompts	Requires prompts and assistance	Unmotivate d

	Cooperation	Fully co-operative	Requires encouragement	Requires directed verbal prompts	Requires prompts and assistance	Non Co-operative
**Other**	Can manage own finances	Independent	Minimal assistance	Moderate assistance	Maximum assistance	Unable
	Formal care package	None needed independent	Small appropriate package in place	Large appropriate package in place	Inappropriate package	Requires care
	Informal carers (family/friends)	Independent but has reg. contact	manages with min support from them	Manages with moderate support	Carer(s) under stress	Carer(s) unable to continue
